# The Development of Maternity Services in Bristol
*An Address to the Midwives of the Bristol Area


**Published:** 1973-10

**Authors:** C. C. Hancock

**Affiliations:** sometime Chief Executive Officer of the Southmead Group Hospitals


### The Development of Maternity Services in Bristol*

By

**C. C. Hancock, M.A., D.P.A., F.H.A.**

sometime

Chief Executive Officer of the Southmead Group Hospitals

The history of the development of the maternity services in the Bristol Area since 1948 would not be complete without the story of the service before 1948.

Having been closely associated with the development of the maternity services with the Bristol Local Health Authority, under Bristol's first full-time Medical Officer of Health, Dr. D. S. Davies, then later Dr. R. A. Askins, followed by Professor R. H. Parry and later Professor R. C. Wofinden, I have, therefore, seen the remarkable progress in the health services, which collectively resulted in the outstanding reduction in maternal and infant mortality, and the improvement in the health of mothers and babies, which without our unique system of midwifery, I venture to say, would not have been possible.

We have now arrived at the present system of the National Health Service, including Hospital Authorities, University Medical Schools, Executive Councils and Local Health Authorities, but by 1974 a different pattern is likely to emerge, which it is hoped will unify the various branches, with consequent improvement in the public service.

It may be helpful to present the history briefly to you in two sections:—

(1) The period under the Local Health Authorities from the Maternity Act of 1918 to 4th July, 1948; and (2) The period following the implementation of the National Health Service Act on the 4th July, 1948, when, in this region, under the able Chairmanship of Dr. H. G. Tanner, I was given almost a free hand to develop those services which came within my view in the Bristol Area, centred on Southmead Hospital, which was ready for expansion, largely due to the foresight of the Bristol Local Health Authority, guided by Dr. R. H. Parry.

**First Period 1918 - 1948**

When I left St. Peter's Hospital in 1921 (having been there when Southmead Hospital was planned) and joined the staff of the Bristol Health Department, the development of the maternity services under the permissive powers of the Maternity and Child Welfare Act was beginning to take shape. Apart from my personal recollection, I am indebted to the Annual Reports of the Bristol Medical Officers of Health for verification of the various steps taken.

A Miss Richards, who was appointed in 1914, was the Superintendent Health Visitor and Deputy Inspector of Midwives under Dr. J. C. Heaven. There were 20 Health Visitors who were almost always entirely confined to Maternity and Child Welfare work and there were 72 midwives; 18 in maternity homes and 54 in private practice. Many of the midwives were without training qualifications. There were only four municipal ante-natal clinics with 368 mothers attending. Milk grants up to a total of £2,000 per annum were made, details of which had to be submitted to His Majesty's Inspector. There were also 20 voluntary schools for mothers, in the running of which the late Mrs. Wethered, wife of Judge Wethered, and Miss Wethered took a leading part.

There were homes for unmarried mothers—Grove House at Redland; The Bristol Maternity Hospital at Southwell Street; Marlborough House at Kingsdown; and the Salvation Army Hostel. There were maternity institutions for normal and abnormal cases, including Brunswick House with 19 beds for married women only. The Bristol General Hospital had 12 beds, the Bristol Royal Infirmary 10 beds, which later increased to 30, and the Poor Law Eastville Institution had 18 beds. Southmead Hospital as we now know it was not then in existence.

It is unnecessary to quote a large series of statistics showing the reduction in maternal and infant mortality to the present rates but it is worth noting that in 1919 the births notified in Bristol were 6,940, mostly home confinements, the birth rate being 18.44 per thousand population and infant death rate 83.37 per thousand births. It is said that statistics can prove anything, and recently I read in Sir Alec Douglas Home's memoirs a story of two statisticians talking together, who decided that as they already had four children each, they would not risk having any more themselves as every fifth child born in the world was a Chinese!

Between 1921 and 1928 there was a gradual development of the Local Health Authority maternity services, the late Professor Rayner and Mr. R. Statham contributing in an advisory capacity thereto, emphasising that the 'normal tended to become the abnormal' In 1923 it was reported that no midwives were now subsidised by the Local Authority and that midwives' fees were paid wholly, or in part in six cases only during the year. In 1925 midwives sought medical help in only **168** cases and practice by unqualified

**An Address to the Midwives of the Bristol Area*

midwives continued. The City had the use of **1 bed for 6 months per annum** at the Bristol Maternity Hospital, Southwell Street.

The Public Health notifications of Puerperal Fever and Puerperal Pyrexia Regulations came into force in 1926, and I remember the Medical Officer of Health closed the Maternity Unit at the Royal Infirmary because of puerperal infection. The Midwives and Maternity Homes Act 1926 placed additional responsibility on the Local Authority, and on the retirement of Dr. D. S. Davies as Medical Officer of Health in 1928, Dr. R. A. Askins, Chief School Medical Officer, was appointed to the dual post and Dr. Heaven retired, being succeeded by Dr. M. G. Hughes full-time on the 1st June, 1928. This marked the commencement of a new era in development of the Bristol Maternity Services. In that year 27 mothers died in Bristol in connection with childbirth, eight of these from puerperal fever, and 365 babies died under the age of one year, this being 59.8 per thousand births. Dr. Askins outlined a scheme for a Central Health Clinic and the City made a contribution for maternity work to the Bristol Royal Infirmary.

The re-organisation of the City Health Services was further facilitated at that time by the abolition of the Poor Law Authorities. Southmead Hospital, with its small Maternity Unit, under Dr. Percy Phillips, was put under the authority of the Health Committee, Bristol being one of the few towns to take advantage of the permissive powers to constitute a Public Health Hospital.

Dr. Askins left Bristol shortly afterwards having been appointed Chief Medical Officer for Southern Rhodesia, and his Deputy, Dr. R. H. Parry, at the early age of 34 was appointed Medical Officer of Health in 1930.

On his accession in 1931, Dr. Parry took every possible step to ensure the Bristol Health Authority took advantage of the new legislation to stop untrained and handy women doing midwifery, to establish Maternity and Child Welfare Clinics and employ Nurses and Doctors. He secured the co-operation of all the well-known Obstetricians in Bristol, including Mr. D. C. Rayner, Professor H. J. Drew-Smythe, Mr. H. L. Shepherd, Miss Mabel Potter, and Mr. F. J. Hector.

He reported that the social conscience was already awakened to the sad maternal and infant mortality rates and that amongst the many societies formed there were the National Society for the Prevention of Infant Mortality; The National Society for Women; Schools for Mothers Association; and The Care of Unmarried Mothers. Some people thought the mortality rates were due to the mother bearing too many children and had started birth control clinics, one being established at Redcliffe Hill.

During the ten years up to the outbreak of war an impressive programme of Health Centres, linked with the new Central Health Clinic in Tower Hill, was planned and partially implemented. Joint appointments of full-time and part-time medical officers for Maternity and School Medical work were made. The municipal district maternity service was established and linked with the training of midwives.

Briefly, the following are some of the developments which took place during the period mentioned :

In 1930 although Southmead became a Bristol City Hospital, an agreement was made whereby maternity cases were also admitted on behalf of the Gloucestershire and Somerset Health Authorities. An elaborate system of assessment of patients' ability to pay was inaugurated, milk and similar foods were provided and a full-time Inspector of Midwives appointed. X-rays were first arranged for maternity patients, Mr. Statham was appointed to Southmead Hospital and the number of maternity beds increased to 44. In 1934 sterilised maternity outfits were supplied at cost price, and in the following year Portway Joint Clinic was opened, the Central Health Clinic opened with specialist services and the Bedminster and Speedwell Clinics were under construction.

In 1937 a scheme under the Midwives Act for an adequate Midwifery Service was implemented; the Central Health Clinic was opened with specialist services, the establishment of day and night nurseries was agreed and a scale of fees for the services of municipal midwives was put into operation. Dr. Margaret Hughes' survey (published in the 1938 M.O.H. report) stated that confinements during that year at home by doctors were 823, by midwives 2,090, and in hospitals 4,049. All this was under the shadow of war preparations and consequent local considerations, the war breaking out in September 1939.

In the mid-thirties the Bristol Royal Infirmary gave notice that as maternity work was the responsibility of Local Health Authorities and the Infirmary's commitments for general work were heavy, they could no longer provide institutional maternity beds. As these beds were then also the main obstetric training ground for medical students, Dr. Parry soon had plans prepared and a scheme accepted by the Council for a large maternity unit with 100 beds to be built at Southmead Hospital, together with an Out-Patient Department, Casualty Section, Maternity, Child Welfare and School Medical Clinic, a new Nurses' Home and Midwifery School Buildings. The University welcomed the step in the interests of training of doctors and midwives. The buildings were completed and opened soon after hostilities commenced in 1939/40. Thus Southmead Hospital became the main centre for training undergraduates in midwifery, post-graduate training of doctors and also the only 'Part I' school for the training of midwives and also the Midwives Teachers' Course.

In connection with the planning of these buildings, the following interesting points arose. Mr. Laurence, the architect, suggested to the Planning Authority that the access should be from a new road to be called Monks Park Avenue, then under construction, and this was agreed. The Ministry decided upon chalet type buildings, apparently for two reasons; the problem of infection — antibiotics as we now know them not being available, and also because, as the war threatened and the site was near the large aeroplane factories, it was desirable for the buildings to be spread out. The Ministry also agreed to an air raid shelter being constructed under the Casualty and Out-Patients Department. As is now known the first large daylight raid on the Bristol area was aimed at Filton and a large number of casualties were brought to Southmead Hospital. Also during an air raid a number of bombs straddled the Hospital itself but in each case landed between the spread out buildings and not a single casualty occurred.

The war brought about a rapid production of new antibiotics, which revolutionised the art of medicine. The war and air raids brought many problems for the maternity services, including the opening of the evacuation nurseries, the maternity beds at Cedar Hall, Frenchay, and at Mortimer House, Clifton, where a 'second part' Midwifery Training School was set up. The midwives in the maternity hospitals and homes, together with the district midwives rendered yeoman service during the war period.

From 1944 - 1948, although it was clear that a National Health Service would come into being, the Local Authority, guided by Professor R. H. Parry, did not hold up progress. Indeed, I was credibly informed that the late Mr. Aneurin Bevan said that if all Local Authorities had been as progressive as Bristol a different pattern of National Health Service might have emerged.

In 1944 Dr. Parry prepared a scheme for the care of premature babies at Southmead Hospital and gas and air analgesia was introduced for district work. The care of illegitimate children was reviewed and in 1945 a comprehensive plan for the provision of district health centres surrounding the Central Health Clinic was prepared. As regards the Premature Baby Unit, Dr. Beryl Corner played a leading part in this new venture. In 1946 residences were acquired for district midwives and 'rhesus' tests were commenced in conjunction with the Blood Transfusion Centre.

### The Second Period 1948 - 1973

On 4th July, 1948 the National Health Service Act was implemented. This was a revolution rather than an evolution in the medical services of the country and the advance of medical knowledge brought about what was termed as a 'medical explosion' and this also applied to the obstetric world.

The following is a brief description of the maternity services with which I, as the Group Administrator, was closely associated.

The Local Voluntary Hospitals, when nationalised, were already overloaded and could not accept any expansion of work, but thanks to the City Fathers and Professor Parry, Southmead Hospital with its new buildings and ample space was ready to develop as a full general and maternity hospital, and Alderman J. J. Milton, the first Chairman of Southmead Group Management Committee, instructed me to prepare a plan for its rapid expansion and introduce all the new departments necessary to meet the new situation for Bristol and its environs.

So far as the maternity services were concerned the great change was the fact that every woman (and child) was now registered with a General Practitioner, and entitled to a free midwifery service. Thus a new era opened for co-operation between the midwives and the General Practitioners who soon realised the key position which the midwives held. There was the complication that many General Practitioners did not wish to attend midwifery cases (although they were paid special fees if they did so), and alternative arrangements had to be made accordingly. Social conditions also had changed, causing problems for home confinements and the consequent increased demand for hospital confinements. The main responsibility for midwifery, and training of midwives, for what was called the Bristol Clinical Area—i.e., Bristol, South Gloucestershire and part of North Somerset, devolved on the Southmead Group, including co-operation with the University in the training of medical undergraduates in obstetrics. The Bristol Maternity Hospital in Southwell Street was later transferred to the former Convalescent Home on the Downs, and continued to function under the able supervision of Miss Norah Dean the Matron and included a 'Part II' School for Midwifery training. Otherwise all the other maternity units in the area, from Berkeley to Clevedon, came under the jurisdiction of my Hospital Authority, including the comprehensive maternity and paediatric departments at Southmead, Mortimer House, Wendover Maternity Hospital at Downend, Berkeley and Almondsbury Hospitals. The pressure for institutional confinements continued and St. Brenda's Hospital, Clifton, was acquired for a General Practitioner Maternity Unit, the first in an industrial town. The Knoll at Clevedon (1951) came into use for the same purpose.

In 1952 a new ward under the care of Dr. Beryl Corner and Professor A. V. Neale was opened at Southmead Hospital for 28 infants under one year, and 12 beds for well mothers with babies. In 1966 a New Maternity Unit with out-patient sessions was opened at Thornbury Hospital (20 beds), the Walker Dunbar Hospital, Clifton began to take maternity cases and contractual arrangements were made with the Salvation Army Home for the admission of some maternity patients.

The following are some of the interesting problems solved, and developments which have taken place since 1948.

#### The Emergency Admissions Bureau

An Emergency Admissions Bureau was established at Southmead for the whole of the Bristol area. It was closely associated with the Group Admissions Department, which had a separate section to deal with maternity admissions and applications for the area. These varied between 8,000 - 10,000 per annum, with all the problems involved, cancellations and the increasing number sent in for a variety of reasons, as emergency admissions. Criteria for hospital delivery and admission to General Practitioner units respectively were prepared and detailed arrangements for planned early discharges were agreed with the Bristol, Gloucestershire and Somerset Health Authorities. The Central Midwives Board were alarmed at first with the turn of events, but eventually moved with the times. There is still a reluctance to use the G.P. Units to the fullest extent and changes will no doubt emerge with the opening of the new maternity unit on St. Michael's Hill.

In connection with the Bureau I would like to pay a special tribute to the late Dr. H. M. Golding and Mr. Ralph Batten.

#### The Obstetric Flying Squad

An Obstetric Flying Squad, based at Southmead Hospital, was established for the area in 1952, details being circulated to all doctors and midwives in Bristol, Somerset and Gloucestershire. Normally the Flying Squad would be called by the General Practitioner but in an emergency the midwife was authorised to do so. The advantages of the Blood Transfusion Service being situated at Southmead was here again emphasised.

**Premature Babies**

The care of premature babies in the Bristol area is a story in itself and commenced with the adaptation of a labour ward in "A" Block at Southmead Hospital. On the request of Professor Parry, I well remember visiting Southmead with Dr. Corner, to discuss with the late Dr. Phillips the implementation of this project. The Southmead Hospital Management Committee improved and developed the service with the full co-operation of the Local Health Authorities of the area (Bristol in particular), and the General Practitioners. It is an outstanding example of the kind of co-operation which can occur in the National Health Service and which proved so beneficial to all concerned. Later, a small unit was also established at the Bristol Maternity Hospital.

1948 saw the birth of the Good quads at Southmead, the first to survive in this country for 125 years. We have heard of their progress from time to time. As usual with pioneer efforts we were the last to acquire a new modern unit, but before I retired, after strong representations, a new comprehensive Special Care Baby Unit was under way and is now in use.

The Premature Baby Unit Scheme was extended to cover the area from Berkeley to Weston, and a baby flying squad team inaugurated, designed to provide immediate resuscitation for premature infants born at home, and to give special care to those more robust infants who were fit to remain at home. The Local Health Authority also provided a domiciliary nursing service for the purpose.

Associated with the care of premature babies, a Human Milk Bureau was established at Southmead in 1950. This scheme was sanctioned by the South Western Regional Hospital Board, and covered the whole region. A small Working Party worked out all the problems involved with equipment, staffing, collection, sterilisation and pasteurising, distribution, and day to day administration, including payment to domiciliary donors. The scheme soon became known and requests for supplies were received, even from outside the region.

**The Blood Transfusion Service**

The Government Regional Blood Transfusion Service has made a special contribution to the maternity and baby service and at Southmead in particular. Established at Southmead Hospital with the co-operation of Professor Parry at the beginning of the war (under the direction of Sir Lionel (then Col.) Whitby), it was the first to be inaugurated in Europe and out of it developed the National Blood Transfusion Service and the S.W. Regional Unit now in first class new buildings under the direction of Dr. G. H. Tovey. Incidentally Whitby was one of Dr. Parry's students when he was Casualty Officer at the Middlesex Hospital. Naturally when he was asked to establish the Army Blood Transfusion Service he thought of Bristol and discussed the project with Dr. Parry. It was agreed that in return for Dr. Parry taking his case to the Bristol Health Committee he would provide a free service to the civilian population of the South West. Dr. Parry and the late Prof. Hey Groves made the first appeal for volunteers on the radio. The British Red Cross co-operated and I was authorised to entertain to luncheon the organisers each year on behalf of Dr. Tovey. All patients, public or private, receive the benefit of this service free of charge.

There is no need for me to detail to you the great saving of the lives of babies and mothers which has been achieved and now we have the transfusion of babies, even before they are born! There was the discovery of special blood groups and the introduction of the card which should be carried at least by every prospective mother, showing her blood group.

**X-ray and Laboratory Services**

The development of the X-ray diagnostic services is a fascinating story, very special arrangements were made by Dr. G. R. Airth for x-raying mothers, babies and the transfusion of babies before birth in co-operation with Professor Gordon Lennon.

The extraordinary developments in the laboratory service have been invaluable in all aspects of the care of maternity patients and babies and much clinical research has been, and is still, undertaken in the Southmead laboratories under the direction of Dr. F. J. W. Lewis and Dr. Norman Brown. Before Dr. Peter Dunn went to Canada I was instrumental in providing him, albeit unofficially, with certain research facilities at Southmead and I am pleased to see he has now returned to the staff here.

**Educational Activities**

Southmead became the centre for training medical students in obstetrics and paediatrics, the training of midwives, refresher courses, the Midwives' Teachers' Certificate, refresher courses under the C.M.B. rules, the training of Consultants in Obstetrics, refresher courses for General Practitioners and the training of general nurses in obstetrics.

There was a continuous review of the training arrangements for midwives in co-operation with all concerned, adjustments made from time to time, special visits being made by the Secretary, Mr. Fenney of the Central Midwives Board, particularly as Southmead was an examinations centre. I enjoyed the visits of the examiners, and entertained to luncheon many well-known personalities in the obstetric world, including Sir John Peel, Professor McClaren and the Earl of Cranbrook of the famous Cranbrook Report.

It was a notable occasion when the C.M.B. officially approved the lying-in period being reduced from 14 to 10 days!!! A block system of training was in being and when I retired the C.M.B. was asked to approve a ten months course of combined midwifery training. Women were allowed to have their husbands present, at least up to the first stage of labour, subject to suitable conditions.

Thus the Southmead Group became a centre for midwifery for the area, the only other but important unit being at the Bristol Maternity Hospital. The confinements in the Group increased from about 4,500 in 1949 to over 8,000 in 1970 when there were no maternal deaths.

In Professor Wofinden's excellent report for 1969, the birth rate was 15.6 per 1,000 population and the illegitimate births 686. The infant mortality rate was 16.2 per 1,000 the lowest recorded, and the second lowest of the 12 large towns. The General Practitioner short-stay unit was recorded as being a success, birth control advice continued to be given and there was co-operation with the hospitals for cervical smear tests. Dr. Sarah Walker stated that the births registered in Bristol were 6,462, full co-operation being maintained with regard to planned early discharges and the home confinements were 646.

It was Sir Winston Churchill who said — "To understand the present and plan for the future you must study the past". It is 53 years since I became aware of the maternity problems at St. Peter's Hospital. Perhaps one of the most striking changes to me is the outlook towards, and the care of, unmarried mothers. I was actually present at a small Committee in 1918/9, including a magistrate, when the legal representation was made that an unmarried mother with two or more illegitimate babies was a moral mental defective, was so certified and sent to Stapleton (now Manor Park) Hospital. The problems of family planning, contraception and abortions now splash the headlines.

There will no doubt be radical changes in 1974 in regard to the administration of the Health Services and the responsibilities of the Local Health Authorities—one can only hope that these changes will be for the better. I have been associated with three such major changes in the past. These changes will not affect the professional role of the midwife, who, I believe, will continue to play a most important part in the maternity services. There may be a hard core in maternity care which cannot be tackled, as in the final analysis, the problem is an economic and financial one. The same kind of problem arises in relation to such projects as heart transplants, and perhaps to a lesser extent with kidney transplants and artificial kidney machines. During my twenty years as Administrator of the Southmead Group, I received medical and nursing visitors from all parts of the world, including 23 doctors from behind the Iron Curtain, who all spoke with envy of our maternity hospitals, the midwifery service and our unique free Blood Transfusion Service.

